# Autonomous learning and creative cognition: the mediating effect of gifted students’ self-efficacy

**DOI:** 10.3389/fpsyg.2024.1301528

**Published:** 2025-01-03

**Authors:** Şenol Orakcı

**Affiliations:** Department of Educational Sciences, Aksaray Faculty of Education, Aksaray University, Aksaray, Türkiye

**Keywords:** self-efficacy, autonomous learning, creative cognition, gifted students, Turkey

## Abstract

**Introduction:**

In today’s world, it is of great importance to raise qualified learners whose creative thinking skills and self-efficacy are developed and who can make various choices and take responsibility for their choices as well as implementing them by making their own decisions. In this regard, the study examined the role of gifted students’ self-efficacy (SE) as a mediator on the relationship between autonomous learning (AL) and creative cognition (CC).

**Methods:**

A proposed conceptual model was tested using a cross-sectional survey design. Based on “convenience sampling,” the study group consisted of 528 gifted secondary school students enrolled in three Science and Arts Centers in Ankara, Türkiye in the 2022–2023 academic year. A two-step Structural equation modelling (SEM) analysis was conducted for data analysis.

**Results and discussion:**

The findings revealed that AL positively and significantly predicted CC, SE was positively correlated with CC, and both dimensions of AL (Independence of Learning- IoL and Study habits- SH) had a significant direct and indirect influence on CC via SE, confirming that the dimensions of AL (IoL and SH) had distinct indirect influences on CC via SE. The study improves our understanding of the role that SE beliefs play in the interaction between AL and CC, which helps to expand and improve models that represent these processes. In order to create conditions that encourage AL, foster SE beliefs, and eventually improve CC among gifted students, educators, policymakers, and parents can create a learning environment that not only promotes AL and strong SH but also fosters SE and CC, ultimately leading to more innovative and self-reliant students.

## 1 Introduction

Today, the focus of education is not knowledge, but the learner. The learners effectively should construct their own knowledge based on their prior knowledge. In other words, today’s learners should create and reorganize knowledge individually. In a learner-centered system, individuals’ active participation in the learning process and making sense of their own knowledge structures are of great importance. For learners to create their own knowledge, they need to move away from the role of the receiver of the information in the traditional classroom, work in an effective communication with the teacher and other friends, and assume responsibility for their own learning, which is the basic feature of “learner autonomy.” In fact, in line with the changes in the sphere of education in the recent past, the need for student-centered learning, making decisions based on students’ own learning, providing this opportunity to students and supporting learners in this direction instead of teacher-centered learning approaches in learning environments has increased. While Holec (1988) defines “learner autonomy” as the ability of the learners to make their own decisions, in short, to take responsibility for learning, in making decisions about all aspects of learning at the stages of setting goals, defining the content, choosing the methods and techniques to be utilized, and assessing what will be achieved, Little (1999) defines it as organizing learning environments by taking into account individual differences and using appropriate strategies. From this point of view, “autonomy” is crucial because it allows the learner to be in control of his/her own learning instead of being under the control of someone else (Liu, 2015). Benson (2011) defines “autonomy” as being responsible for one’s own learning. “Learner autonomy” is also regarded as the first condition of active learning. When learners are successful in developing their autonomy, they also become responsible individuals in addition to being a good learner (Benson, 2011). Therefore, the learner himself/herself should make some decisions that shape the learning process, should supervise their development and participate actively in this process in evaluating which learning goals have been achieved (Little and Dam, 1998).

Individuals who can make up their own mind up about learning and shape their learning can also make their own decisions about any subject in other dimensions of their lives. Autonomous learners have seven main characteristics as learners (a) who are tolerant and have warm approach to what they learn, (b) who are aware of their own learning strategies and styles, (c) who try to take an active part in learning, (d) who are willing to take risks, (e) who pay attention to the form as well as the content, (f) who have good foresight, (g) who develop what they have learned in a unique way and who review and eliminate the information they do not use (Thanasoulas, 2000).

### 1.1 Creative cognition (CC)

Creativity is a complicated process that depends on the individual’s way of thinking and many other variables such as “environment,” “culture,” “individual competencies” and “thoughts” have an effect on it (Rogaten and Moneta, 2016; Sligh, 2003). In spite of all these various factors, mental processes are considered to be the essence and motivator of creative effort. Despite the fact that many useful approaches have been suggested to comprehend creativity, the “creative cognition approach” (CCA) is important because it is centered in the cognitive structures and processes that underpin creative thinking. As the concept of creativity develops over time, CCA has been suggested in creativity studies. This approach suggests that creativity is “a multidimensional construct” based on “multiple cognitive processes” (Sligh, 2003). The CCA is concerned with the application of thinking strategies and creative techniques that promote being creative (Davis, 2004; Sternberg and Lubart, 1996). The CCA can be described as “the tendency to propose original solutions and new products” (Gardner, 1988) and “the construction of something that did not exist before” (Roskos-Ewoldsen, 1993). The concept of the use of “creative cognition” (CC) originates from the CCA (Finke et al., 1992; Ward et al., 1999),

The CCA primarily focuses on the “conceptual structures” and “cognitive processes” used in the generation of creative ideas. Its objectives are to benefit from the experimental and theoretical developments of cognitive science in comprehending creativity and to utilize creative performance as a way to learn more about basic “cognitive processes.” Guilford (1967) was also the first to introduce the CCA.

The CCA aims to give supplementary strength to the basic cognitive research of creativity. This approach considers the significance of other important “non-cognitive” factors such as “motivation,” “personality traits,” “formal education,” “counseling,” and other “social and historical forces” (Csikszentmihalyi, 1997; Simonton, 1999) that affect creativity. However, its focus is on the cognitive processes involved in the generation of new and useful ideas, which are the basis of creative developments in practice. In other words, the likelihood and frequency of individuals being involved in cognitive functions, the information provided to the creative individual, and the likelihood of a newly generated idea being accepted should clearly be influenced by the other factors mentioned. Therefore, a full explanation of human creativity requires specifying the interaction of such factors.

### 1.2 Self-efficacy (SE)

Self-efficacy, a key element of Bandura's (1997) social learning theory, reflects one’s belief in one’s ability to perform a task successfully (Bandura, 1988; Bong, 2004; Chen et al., 1998). This belief relates to an individual’s self-assessment in terms of how they anticipate they will respond to a specific situation or set of circumstances they may face (Britner and Pajares, 2006; Chen et al., 2004). According to Bandura, the crucial factor is an individual’s belief in their ability to utilize their skills (Bandura, 1997). Self-efficacy is the subject of extensive research and publications in various fields, such as education, medicine, psychology, business administration, and international relations, confirming its decisive impact on behavior (Bandura, 1986). High self-efficacy beliefs enhance success and personal satisfaction (Bandura, 1994). Individuals with elevated self-efficacy can tackle challenging events or studies with confidence, resulting in a more productive outcome. Conversely, individuals with low levels of self-efficacy perceive obstacles as more daunting. This belief may heighten an individual’s apprehension and stress levels, as well as narrow their outlook on events and issues. An individual’s self-efficacy belief is a significant aspect that influences their perception of success (Pajares, 2002). The self-efficacy level of individuals can either positively contribute to or impede motivation. For instance, individuals with a high sense of self-efficacy opt for more demanding tasks, which places them in a more arduous predicament and leads to self-inhibition (Bandura, 1997). Additionally, it is asserted that optimistic self-efficacy expectations enhance motivation, enable individuals to overcome challenges in any novel task encountered and result in greater diligence. Negative self-efficacy expectations, however, are believed to inhibit an individual’s ability to take initiative in tasks they undertake or lead to incomplete task completion (Jerusalem, 2002).

### 1.3 The link between autonomous learning, creative cognition, and self-efficacy

It is emphasized in both theoretical and applied research that autonomous learning and creative cognition skills directly or indirectly affect positively each other. However, very few studies have attempted to investigate this relationship between them. Of these studies, Shemirani et al. (2011) in their research, examining the relationship between autonomy, creativity, and language proficiency, concluded a positive and significant relationship among the level of autonomy, creativity and language proficiency. The findings also revealed a clear link between autonomy and creativity among students. In their study, Nosratinia and Mirzaki (2014, 2015) examined the relationship between creativity and autonomy among learners, and revealed a significant and positive relationship between the creativity and autonomy of learners. Nosratinia and Mirzaki (2014) in their study revealed that creative thinking can significantly contribute to the degree of autonomy among learners. Hashamdar and Rangriz (2017) also concluded a significant relationship between learners’ autonomy and creativity skills. Similarly, Çelik (2017) revealed that the concepts of autonomous learning and creative cognition were interrelated, and that creative cognition positively predicted autonomous learning and its sub-dimensions, “independent learning” and “study habits.” Additionally, Hermita et al. (2023) found that an autonomous learning application in STEM classes improved students’ critical thinking abilities in their quasi-experimental research. To put it another way, encouraging critical and creative thinking can help them become more autonomous since autonomous learning requires critical and creative thinking and behavior as well as correct judgment based on interactions, experiences, and observations when analyzing, organizing, and evaluating information.

A study by Demir and Cetinbas (2023) revealed that critical thinking was a strong predictor of autonomous learning in the regression analysis. Highly capable students’ autonomous learning is impacted by any shift in critical thinking (Demir and Cetinbas, 2023). Another study by Arslan et al. (2023) also showed that autonomous learning significantly predicted both problem-solving and creative cognition. According to Winch (2002), autonomy is one component of psychological well-being that can influence students’ learning outcomes. Students with strong degrees of autonomy will also have strong levels of learning independence. People with high levels of psychological well-being are content, healthy, productive, and have fulfilling interpersonal interactions (Ryff, 2013), indicating the likelihood of a relationship between autonomous learning and creative cognition. Given these explanations and findings between learner autonomy and creative cognition, this paper hypothesizes the following:

*Hypothesis 1:* “Autonomous Learning” will be positively correlated with “Creative Cognition.”

Tabassam and Azhar (2021) found out a positive correlation between learner autonomy and self-efficacy. He states that learner autonomy and self-efficacy are closely related, because when students are aware of their potential, they are better able to perform their tasks. Tılfarlıoğlu and Çiftçi (2011) found that students with high self-efficacy from Gaziantep, Zirve, İnönü, Selçuk and Karatay Universities in Türkiye were more likely to be autonomous in the language learning process because learners’ beliefs about their abilities in the language learning process affected how autonomous they were. According to Cotterall’s (1999) research, students’ self-efficacy and their actual performance in autonomous learning are significantly related. Cotterall (1999) investigated the main determinants of learners’ beliefs and identified students in her study who had strong self-efficacy in relation to the characteristics that autonomous learners would exhibit. In other words, autonomous learning can be predicted by students’ confidence in their ability to learn English and to do more. Cotterall (1999) found that even when students did not master these tactics, those with strong beliefs exhibited more autonomous behavior when using various strategies, including goal setting and organizing their English learning processes. Ilgaz (2011) concluded in his study that self-efficacy perceptions significantly predicted autonomy perceptions. Self-Directed Learning (SDL) states that people are inherently driven to learn new things and take on challenges, and that they have a natural tendency to acquire autonomous regulation of behavior (ten Cate et al., 2011). Common definitions of SDL include students’ capacity and disposition to create, pursue, and assess their own learning goals as well as their ability to assess their own learning process and outcomes (Abd-El-Fattah, 2010; van Woezik et al., 2021). It has been shown that self-directed learning helps learners improve their autonomous thinking, reflection, and creativity. SDL indicates more autonomous learning practices and gives students greater control over their own education (Kemp et al., 2022). A significant and positive correlation between the dimensions of academic self-efficacy and learner autonomy was found by Dong and Mustapha (2020) among English majors. For example, a study conducted on Hungarian secondary school students by Csizér et al. (2021) showed that autonomy was correlated with better levels of motivation, self-efficacy, and emotional well-being. This suggests that learners who are autonomous are more likely to use self-regulation techniques, create personal learning objectives, and ultimately have better success and fulfillment in their educational endeavors. Based on the established relationship between learner autonomy and self-efficacy, the following hypothesis is proposed:

*Hypothesis 2:* “Autonomous Learning” will relate positively to “self-efficacy.”

Social cognitive theory suggests that high levels of self-efficacy are a necessary (but not sufficient) condition for creative output (Bandura, 1997, 2001), indicating the likelihood of a relationship between self-efficacy and creative cognition. Bandura (1997) also emphasizes that “innovativeness largely involves restructuring and synthesizing knowledge into new ways of thinking and of doing things… an unshakable sense of efficacy (is required) to persist in creative endeavors” (p. 239). According to Bandura (2001), individuals will not invest time, money and effort in creative endeavors unless they believe in their ability to produce original results. Tierney and Farmer (2002) extended Bandura's (1997, 2001) theory to creative self-efficacy by arguing that creative self-efficacy facilitates creative outcomes through a series of actions such as (1) attempting creative tasks, (2) investing effort in the creative process, and (3) maintaining persistence in the face of challenges (Du et al., 2018). Researchers and educators find it interesting to examine how learners’ self-efficacy affects their creative capacity (Beghetto, 2006; Byrge and Tang, 2015). According to Song et al. (2022), there is a clear correlation between SDL and problem solving ability, which is related to critical thinking in the field of education. Meanwhile, critical thinking and creative thinking have been found to be positively correlated (Liu et al., 2021). Thus, creativity and SDL can be linked. The results of studies conducted by Lunyk-Child et al. (2001), Zhoc et al. (2018), and Shafait et al. (2021) indicated that SDL had a favorable correlation with a number of learning outcomes, such as increased confidence, intrinsic drive to learn, critical thinking, and creativity. In fact, numerous studies have been conducted on the relationship between self-efficacy and creative thinking skills (Choi, 2004; Farmer, 2017; Gao, 2020; He, 2022; Laws, 2002; Seo et al., 2015). For example, He (2022) in her study revealed that creative self-efficacy substantially explained 11% of the variance in the propensity to use creative cognition, and that the relationship between self-efficacy and the propensity to use creative cognition was of medium size. Furthermore, a meta-analysis of 41 empirical studies examining the relationship between creative self-efficacy and creativity found that the overall mean strength of the relationship was of moderate size (*r* = 0.39; Haase et al., 2018). These findings provide evidence in favor of both the creative behavior and the social cognitive theory. In light of these explanations and the findings between “self-efficacy” and “creative cognition,” this paper hypothesizes the following:

*Hypothesis 3:* “Self-efficacy” will be positively correlated with “creative cognition.”

Students’ autonomous learning attempts is expected to have a greater effect on their capacity for creative thought when they have self-efficacy, or the conviction that they can achieve. Thus, increased self-efficacy is a result of autonomous learning, and this increases creative cognition. A bulk of empirical literature (e.g., Çelik, 2017; Hashamdar and Rangriz, 2017; Nosratinia and Zaker, 2015; Nosratinia and Mirzaki, 2014; Ryff, 2013; Shemirani et al., 2011) has uncovered a positive relationship between learner autonomy and creative cognitive skills. Learner autonomy and creative cognition are expected to be related because people with high levels of psychological well-being are satisfied, healthy, productive and have satisfying interpersonal relationships (Ryff, 2013). On the other hand, empirical literature (e.g., Cotterall, 1999; Ilgaz, 2011; Tabassam and Azhar, 2021; Tılfarlıoğlu and Çiftçi, 2011) has associated learner autonomy and self-efficacy. Tabassam and Azhar (2021) asserts that learner autonomy and self-efficacy go hand in hand, as students are more capable of performing their tasks when they are aware of their potential. Tılfarlıoğlu and Çiftçi (2011) found that students with high self-efficacy were more likely to be autonomous in the learning process, because learners’ perceptions of their abilities in the learning process influenced how autonomous they were. Ismail et al.’s (2023) study on Iranian EFL students discovered that self-efficacy was greatly increased by authentic assessments, which support autonomous learning. By requiring students to apply their information in practical situations, these assessments helped them develop their self-regulated learning skills and strengthen their self-confidence. In a research conducted on Hungarian secondary school students, Csizér et al. (2021) found a correlation between autonomy and increased motivation, self-efficacy, and emotional well-being. This suggests that learners who are autonomous are more likely to use self-regulation techniques, create personal learning objectives, and ultimately have better success and fulfillment in their educational endeavors. A compact review of literature also reveals a positive relationship (e.g., Byrge and Tang, 2015; Du et al., 2018; He, 2022; Seo et al., 2015) self-efficacy and creativity. Self-efficacy and creative cognition are likely to be related, as social cognitive theory postulates that high levels of self-efficacy are a necessary (but not sufficient) condition for creative performance (Bandura, 1997, 2001). Higher education-based research by Odoch et al. (2024) has shown that self-efficacy dramatically improved a variety of creative behavior aspects, including idea development, idea advocacy, and idea execution. The execution phase was where self-efficacy had the most influence, indicating that self-efficacy is essential to realizing innovative ideas. Another study by Newman et al. (2018) found that creative self-efficacy significantly boosts innovative behavior. These results imply that learning practices that support learner autonomy can increase self-efficacy, and that self-efficacy can play an intermediary role in how autonomous learning influences creative cognition. In other words, the effect of autonomous learning on creative cognition is likely to be explained through self-efficacy. Based on the above theoretical and practical research, this paper hypothesizes the following:

*H4:* “Self-efficacy” is a mediator on the relationship between “Autonomous Learning” and “Creative Cognition.”

A strong correlation between independent study habits and creative thinking was found in Butson et al.'s (2020) study of undergraduate health science students. It is more probable that students who participate in SDL are inclined to acquire creative problem-solving skills. Their imaginative application of information and exploration of novel concepts are fostered by these habits, which are essential components of creativity. A creativity-friendly atmosphere is created when independent learning and effective study skills are combined. Research indicates that students are better able to participate in creative thinking and innovation when they acquire effective study habits, such as time management and critical thinking. Self-efficacy also supports creative learning and improves academic performance, as explained by Bidjerano and Dai (2007). Fan and Sun (2024) claim that people with high self-efficacy overcome obstacles, value and enjoy creative solutions, and make full use of their creative abilities to achieve positive results. Self-efficacy was also found to partially mediate the relationship between creative thinking skills and academic achievement by Fan and Sun (2024). Similarly, according to Yan et al. (2022), learning performance was directly impacted by self-efficacy on creativity, which also served as a moderator for the effects of creativity. Overall, fostering independent learning and developing study habits are key strategies for enhancing creative thinking among learners. These practices equip students with the skills necessary for innovative and creative problem-solving. In conclusion, the differential impact of independent learning and study habits on creativity through self-efficacy can show the nuanced ways in which various aspects of autonomous learning contribute to creative outcomes. The reported studies have provided theoretical and empirical evidence that leads the author to suggest that.

*H5:* “Autonomous Learning”dimensions (Independence of Learning and Study habits) are characterized by different indirect effects on “Creative Cognition” through “Self-Efficacy.”

**Figure 1:**
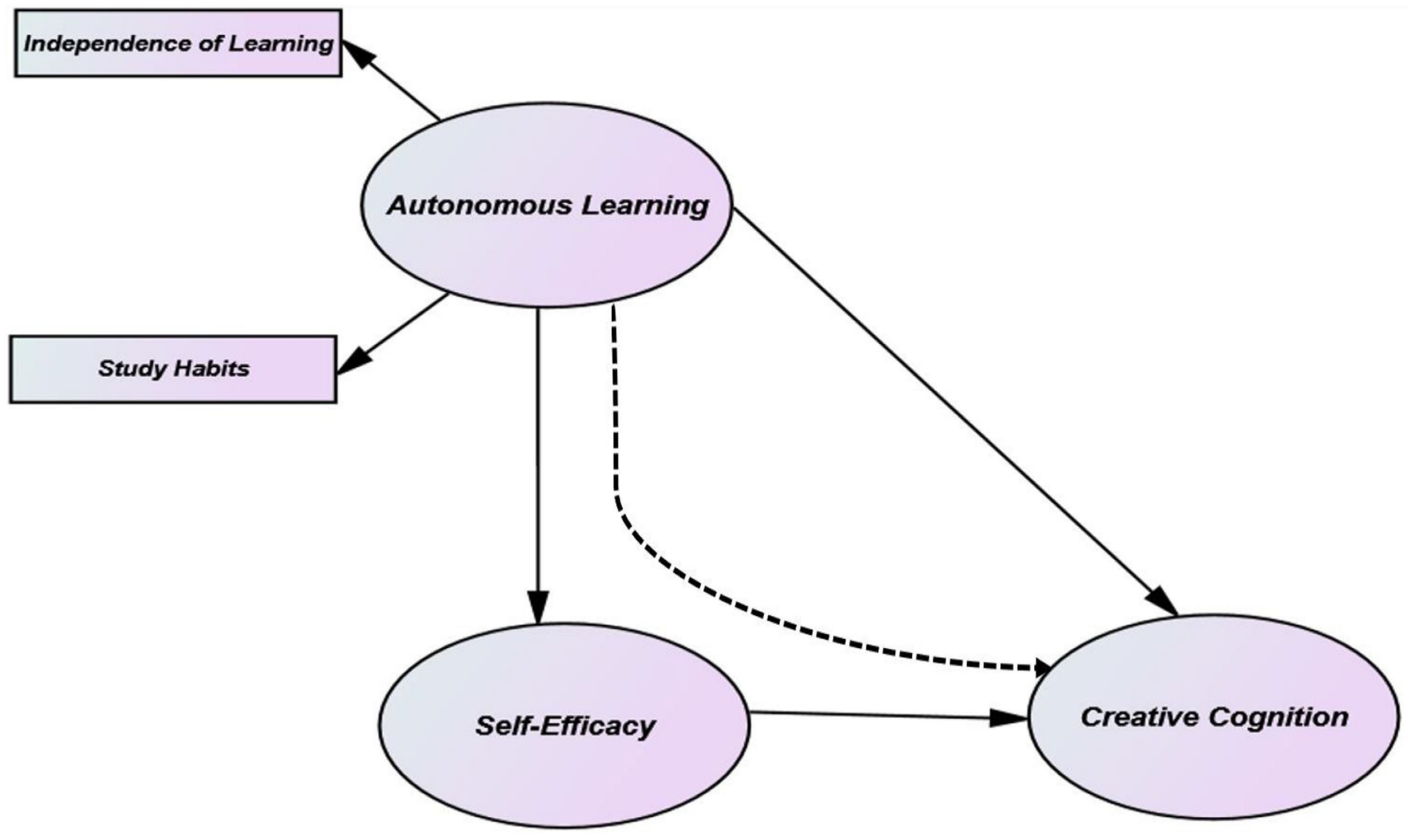
Conceptual framework.

In today’s world, it is of great importance to raise qualified learners whose creative thinking skills and self-efficacy are developed and who can make various choices and take responsibility for their choices as well as implementing them by making their own decisions. Therefore, the current research which is thought to open the door for the field literature is considered to be extremely important. In this regard, the model constructed below (see Figure 1) for the present study represents the hypotheses formulated above.

The research questions corresponding to each of the hypotheses, respectively, are designed as follows:

Is there a positive correlation between “Autonomous Learning” and “Creative Cognition”?
Is there a positive correlation between “Creative Cognition” and “Self-Efficacy”?
Is there a positive correlation between “Self-Efficacy” and “Creative Cognition”?
Does “Self-Efficacy” mediate the relationship between “Autonomous Learning” and “Creative Cognition”?
How do the dimensions of “Autonomous Learning” (Independence of Learning and Study Habits) indirectly affect “Creative Cognition” through “Self-Efficacy”?

Education is changing quickly, and students’ creative cognition and autonomous learning skills are becoming increasingly important. For gifted students, who are frequently expected to thrive in both academics and the arts, this change is especially important. The processes by which autonomous learning affects creative cognition, particularly with regard to gifted students, are still not well understood. Educational results and psychological resilience are significantly influenced by self-efficacy, or the conviction in one’s own capacity for success. Knowing how self-efficacy affects the link between autonomous learning and creative cognition is crucial for gifted students, since they already have advanced cognitive ability. The instructional tactics and interventions designed to help these students reach their full potential can be greatly improved by this understanding.

The complex interactions between autonomous learning and creativity and how self-efficacy influences them may not be adequately addressed by current educational methods and regulations. Studying the interactions between these variables is vital given the growing expectations placed on students to be self-directed and innovative in order to provide the best learning environments and results. Our work intends to fill in current gaps in the literature and offer useful insights for academics, educators, and policymakers by examining this mediating influence. Through a more nuanced knowledge of how to support and enhance gifted students’ learning experiences, our research will help ensure that gifted students may fully utilize their gifts in both academic and creative realms.

### 1.4 Gifted students in the Turkish educational context

Gifted students in the Turkish educational context are described as individuals who exhibit exceptional abilities or potential in various domains such as academic achievement, creativity, leadership, and artistic talents. These students often demonstrate advanced cognitive abilities, high levels of motivation, and a propensity for independent and self-directed learning. Gifted students in Türkiye are identified through a combination of standardized tests, teacher recommendations, and psychological assessments. Once identified, they may receive specialized support and opportunities to nurture their talents through Science and Art Centers (BİLSEMs) programs. Türkiye offers various specialized programs and schools designed to meet the needs of gifted students. These programs provide enriched and accelerated curricula, focusing on areas such as science, mathematics, arts, and social sciences. Enrichment activities and differentiated education are essential components of these programs that address the various requirements of gifted students. In conclusion, remarkable skills and potential are acknowledged for talented children in the Turkish educational setting in a variety of fields. Notwithstanding continuous difficulties and the need for reforms, the educational system strives to offer these students opportunity and individualized support to help them reach their full potential.

## 2 Research method

We used a cross-sectional design, a type of observational study, in the present study to analyse data from gifted secondary school students enrolled in three science and art centers in Ankara during the academic year 2022–2023 with the aim of investigating the role of self-efficacy (SE) as a mediator on the relationship between autonomous learning (AL) and creative cognition (CC). Inferential statistical analyses were conducted in the study taking into account the resources (Cole and Maxwell, 2003; Little et al., 2007; Maxwell and Cole, 2007) that provide a solid foundation and further reading on how inferential statistical analyses, particularly SEM particularly SEM, can be used effectively in cross-sectional studies.

### 2.1 Sample (participants)

The study group consisted of 528 gifted secondary school students enrolled in three Science and Arts Centers located in Ankara, the capital city of Türkiye, during the academic year of 2022–2023. Gifted secondary school students from Ankara, Türkiye, were selected for the study because of their great ability to offer insightful information about educational and developmental processes. These students, who stand out for their extraordinary cognitive capacities, inventiveness, and drive, provide a distinct viewpoint that can influence educational procedures and regulations. Although they have unique qualities, a lot of these qualities are universal, therefore the results apply to a variety of situations. Participants were chosen through the use of “convenience sampling,” a non-probability selection approach in which the researcher chooses participants based on their accessibility and closeness. To reach and interact with particular groups and communities that met the predetermined criteria of the intended audience, online platforms and scales were employed. A request was sent to Arts and Science Centers for assistance in identifying participants. A total of 528 gifted secondary school students volunteered to participate, surpassing the SEM’s minimum requirement of 200 participants (Kline, 2005). They range in age from 11 to 14 years (*M* = 13.1, SD =1.4). 274 (51.90%) of the students were girls and 254 (48.10%) were boys. The number of students in the 6th grade was 156, the number of students in the 7th grade was 219, and the number of students in the 8th grade was 153 in terms of the class levels of the students. The scales were applied in the school setting in May and June 2023. Participation in the study was completely voluntary, and to ensure full and voluntary participation of gifted secondary school students, an anonymous data collection mechanism was implemented. Participants have the option to withdraw from the study at any time. The students were informed of the contents of the scales. It took about 25 min to complete the application process.

Study ethics were approved by the human research ethics committee at the corresponding author’s university. The year and protocol number of the document is 2023/03–03. Consent for the use of the scales was also obtained from the school management and teachers.

### 2.2 Data collection tools

The “Autonomous Learning Scale” (ALS): The ALS developed by Macaskill and Taylor (2010) and adapted into Turkish by Arslan and Yurdakul (2015) was utilized to investigate the learner autonomy levels of the participants. It is a two-dimensional Likert-type scale consisted of 12 items (e.g., “I enjoy new learning experiences,” see Annex for all items). The Cronbach’s alpha reliability coefficient overall was computed as 0.81; 0.80 for “Independence of Learning” (IoL) dimension; 0.80 for “Study habits” (SH) dimension for this study. The scale does not include reverse-scored items and employs a 5-point Likert-type response set. The Turkish adaptation of the scale demonstrated satisfactory to excellent fit for some, but not all, of the confirmatory factor analysis fit indices. Upon analysis of the Confirmatory Factor Analysis (CFA) fit indexes of the ALS as shown in Table 1, it was determined that the model exhibited a perfect or acceptable fit, with the following values: χ^2^/df = 2.63, RMSEA = 0.06, CFI = 0.95, GFI = 0.93, TLI = 0.93, IFI = 0.93, AGFI = 0.94, RMR = 0.04 and SRMR = 0.04.

**Table 1:** Goodness of fit indices.

Constructs	χ^2^/df	GFI	NFI	TLI	CFI	RMSEA	SRMR
AL	2.63	0.93	0.91	0.93	0.95	0.06	0.04
SE	2.67	0.93	0.96	0.96	0.98	0.05	0.04
CC	2.56	0.92	0.97	0.98	0.97	0.04	0.05

The “Creative Cognition Scale” (CCS): The CCS developed by Rogaten and Moneta (2015a,b) and adapted into Turkish by Çelik (2018) was utilized to compute the creative cognition levels of students. The scale with 5 items (e.g., “I find effective solutions by combining multiple ideas,” see Annex for all items) showed one dimension and represent a general creative cognition score. The Cronbach Alpha reliability level of the scale was calculated as 0.75 in the present study. The Turkish adaptation of the scale demonstrated satisfactory to excellent fit for some, but not all, of the confirmatory factor analysis fit indices. When analyzed the CFA fit indexes of the CCS, they indicated a perfect or acceptable fit, with the following values as shown in Table 1: χ^2^/df = 2.56, RMSEA = 0.04, CFI = 0.97, GFI = 0.92, TLI = 0.98, IFI = 0.91, AGFI = 0.92, RMR = 0.07 and SRMR = 0.05.

The “Student Self Efficacy Scale” (SES): The SES developed by Rowbotham and Schmitz (2013) and adapted into Turkish by Özen et al. (2016) to assess students’ self-efficacy levels is a measurement tool consisting of 10 items (e.g., “When I try really hard, I am able to learn even the most difficult content,” see Annex for all items) and one sub-dimension. The Cronbach Alpha reliability level of the scale was calculated as 0.79 in the present study. The Turkish adaptation of the scale exhibited satisfactory to excellent fit for some, but not all, of the confirmatory factor analysis fit indices. The CFA fit indexes of the SES exhibited a perfect or acceptable fit, with the following values as shown in Table 1: χ^2^/df = 2.67, RMSEA = 0.05, CFI = 0.98, GFI = 0.93, TLI = 0.96, IFI = 0.93, AGFI = 0.94, RMR = 0.09 and SRMR = 0.04.

### 2.3 Data analysis

To ascertain whether the data set could be considered to exhibit a normal distribution, the skewness and kurtosis ratios (calculated as the ratio of the skewness and kurtosis values divided by the standard error) were determined as in shown in Table 2 that shows a normal distribution in the present study. Acceptable values for kurtosis and asymmetry between −2 and + 2 are those that show a normal univariate distribution. According to Hair et al. (2014) and Byrne (2016), when the kurtosis is between −7 and + 7 and the skewness is between −2 and + 2, then data is considered to be normal.

**Table 2:** Descriptive statistics and correlation analysis of each research variable.

	Mean	SD	Skewness	Kurtosis	α	AL	IoL	SH	SE	CC
AL	3.976	0.643	−1.182	2.76	0.797	1.000				
IoL	3.967	0.517	−1.191	1.61	0.807	0.879**	1.000			
SH	3.754	0.670	1.142	1.084	0.809	0.798**	0.565**	1.000		
SE	5.798	0.878	−0.376	0.382	0.806	0.869**	0.587**	0.549**	1.000	
CC	3.756	0.601	−1.814	4.879	0.749	0.745**	0.581**	0.531**	0.687**	1.000

*n* = 528. **p < 0.01.

A two-step SEM analysis was conducted for data analysis (Byrne, 2016; Hair et al., 2014). Prior to examining the structural model, which tests research hypotheses, the measurement model’s validity was assessed using IBM AMOS software version 26.0. The measurement model’s convergent validity, discriminant validity, and construct validity were evaluated through confirmatory factor analysis (CFA). The validity of the measurement model was assessed by the researcher using the maximum likelihood estimate on the covariance matrix. Items that had cross-loading or did not meet the intended cut-off threshold were excluded, with a factor loading of 0.60 set as the criterion (Hair et al., 2014). The study’s focal point was in AL, so we presented the findings for the primary variable and its two dimensions (IoL and SH). SE and CC were examined as unidimensional constructs in this study.

To assess mediation theories in the structural model, the bootstrapping analysis method is used to investigate indirect effects (Preacher and Hayes, 2008). Cohen (1992) offers a guide to standardized effect sizes, indicating that results between 0.10 and 0.30 indicate a small effect, while those between 0.30 and 0.60 indicate a medium effect, and those exceeding 0.60 indicate a large effect. The “RMSEA,” “TLI,” “NFI,” “CFI,” and the χ^2^/df value were used to estimate parameters. For a model to be considered as fitting the data satisfactorily, its “TLI,” “NFI,” and “CFI” must exceed 0.90, its “RMSEA” should be less than 0.08, and the χ^2^/df value of it should be <3.0 (Byrne, 2016; Hair et al., 2014).

## 3 Findings

The study aimed to explore the structural models of the proposed connections between the constructs, including Hypothesis 1, where AL predicts CC; Hypothesis 2, where AL predicts SE; Hypothesis 3, where SE predicts CC; and Hypothesis 4, where SE acts as a mediator between AL and CC. The findings begin with validating the model before turning to an analysis of the hypotheses presented in the study.

### 3.1 Measurement model

The correlation coefficients, means, and standard deviations for the study variables are exhibited in Table 2. It has been determined that the Cronbach’s alpha coefficients for internal consistency across the three scales exceeded the proposed standard value of 0.70 by Nunnally and Bernstein (1994). The correlations are significant and align with the expected direction. Based on the descriptive statistics as shown in Table 2, the participants rated all factors as moderate: AL (mean = 3.98; SD = 0.64), CC (mean = 3.76; SD = 0.60), and SE (mean = 5.80; SD = 0.88).

The construct validity of each scale was assessed through a “CFA” analysis conducted using Amos 26.0 software to determine the AL second order and two-dimensional factor structure. The final “CFA” results, as shown in Table 1, indicate a satisfactory fit of the AL two-dimensional model (“χ^2^/df” =2.63, “GFI” = 0.93, “CFI” = 0.95, “NFI” = 0.91, “TLI” = 0.93, “RMSEA” = 0.06) and acceptable fit of the CC unidimensional structure (“χ^2^/df” =2.56), with a “GFI” of 0.92, a “CFI” of 0.97, an “NFI” of 0.97, a “TLI” of 0.98, and an “RMSEA” of 0.04. In Table 3, the results indicate a good fit for the unidimensional structure of SE (“χ^2^/df” =2.67), with a “GFI” of 0.93, a “CFI” of 0.98, an “NFI” of 0.96, a “TLI” of 0.96, an “SRMR” of 0.04, and an “RMSEA” of 0.05. All of the investigated items’ factor loadings were satisfactory, i.e., above 0.60, indicating a good fit.

**Table 3:** Convergent and discriminant validity of the main constructs.

Constructs	CR	AVE	MSV	ASV	AL	SE	CC
AL	0.873	0.772	0.451	0.479	0.897**		
SE	0.921	0.647	0.487	0.457	0.691	0.723**	
CC	0.965	0.651	0.412	0.429	0.797	0.689**	0.735**

*n* = 528. **p < 0.01, Square root values of the AVE are shown in bold with asterisk.

Subsequently, we assessed the discriminant validity and convergent validity of the instruments. We evaluated the convergent validity by analyzing the statistics of the average variance extracted (AVE). The AVE values for the three tools were 0.651 (CC), 0.647 (SE), and 0.772 (AL), as shown in Table 3. All values imply sufficient convergent validity (Hair et al., 2014). To achieve the models’ discriminant validity, the AVE for loaded factors must exceed both the average shared variance (ASV) and the maximum shared variance (MSV) for a model, as shown in Table 3 (Hair et al., 2014). The research findings provide robust evidence of the validity and reliability of the translated instruments employed in the study.

In addition, we applied the Fornell-Larcker criterion (Fornell and Larcker, 1981) in the present study as shown in Table 4 that confirms that the discriminant validity of this analysis is good, as the total AVE square root of the latent variable is bigger than the correlation coefficient of other determinants (Fornell and Larcker, 1981).

**Table 4:** Fornell–Larcker test for discriminant validity test.

	ALS	ALS-IoL	ALS-SH	CCS	SES
ALS	0.787				
ALS-IoL	0.534	0.701			
ALS-SH	0.639	0.579	0.794		
CCS	0.467	0.452	0.423	0.765	
SES	0.465	0.357	0.412	0.376	0.712

Hair et al. (2014) and Wijaya et al. (2022) suggested that the value of the HTMT (Heterotrait-Monotrait Ratio of Correlations) should be monitored in order to thoroughly examine the discriminant validity. When the HTMT value stays below the 0.9 criterion, DV is considered excellent. Table 5 further shows that the greatest HTMT value is 0.829 (PU-TA) using smart-PLS, demonstrating the excellent DV between the latent variables.

**Table 5:** HTMT (heterotrait–monotrait ratio of correlations) values.

	ALS	ALS-IoL	ALS-SH	CCS	SES
ALS					
ALS-IoL	0.629				
ALS-SH	0.713	0.612			
CCS	0.467	0.650	0.529		
SES	0.575	0.343	0.312	0.376	

### 3.2 Structural model estimation

The structural model illustrates the connections between the variables in the theorized model (Byrne, 2016; Hair et al., 2014). The aim of this study is to investigate the relationships between AL, SE, and CC. To accomplish this objective, five hypotheses were formulated. Consequently, two mediation models were assessed: partial and full mediation. The model proposed that there were direct effects of AL on CC, as well as indirect effects mediated by SE (see Figure 1). The full mediation model indicated that all AL effects on CC could be indirect or mediated by SE. Moreover, SEM was employed to compare the results of the full and partial mediation models presented in Figure 1. The partial mediation model (Table 6) achieved an adequate fit for the data (“χ^2^/df” =1.98), with a “CFI” of 0.96, an “NFI” of 0.98, a “TLI” of 0.95, and an “RMSEA” of 0.02. Furthermore, the fit was significantly superior when compared to the full mediation model (“χ^2^/df” = 1.87), with a “CFI” of 0.92, an “NFI” of 0.88, a “TLI” of 0.92, and an “RMSEA” of 0.03, as shown in Table 6.

**Table 6:** Fit indices for partial and full mediation SEM models (*n* = 528).

Model fit indices	χ^2^/df	GFI	NFI	TLI	CFI	RMSEA	SRMR
Partial mediation model	1.98	0.94	0.98	0.95	0.96	0.02	0.02
Full mediation model	1.87	0.93	0.88	0.92	0.92	0.03	0.02

**Figure 2:**
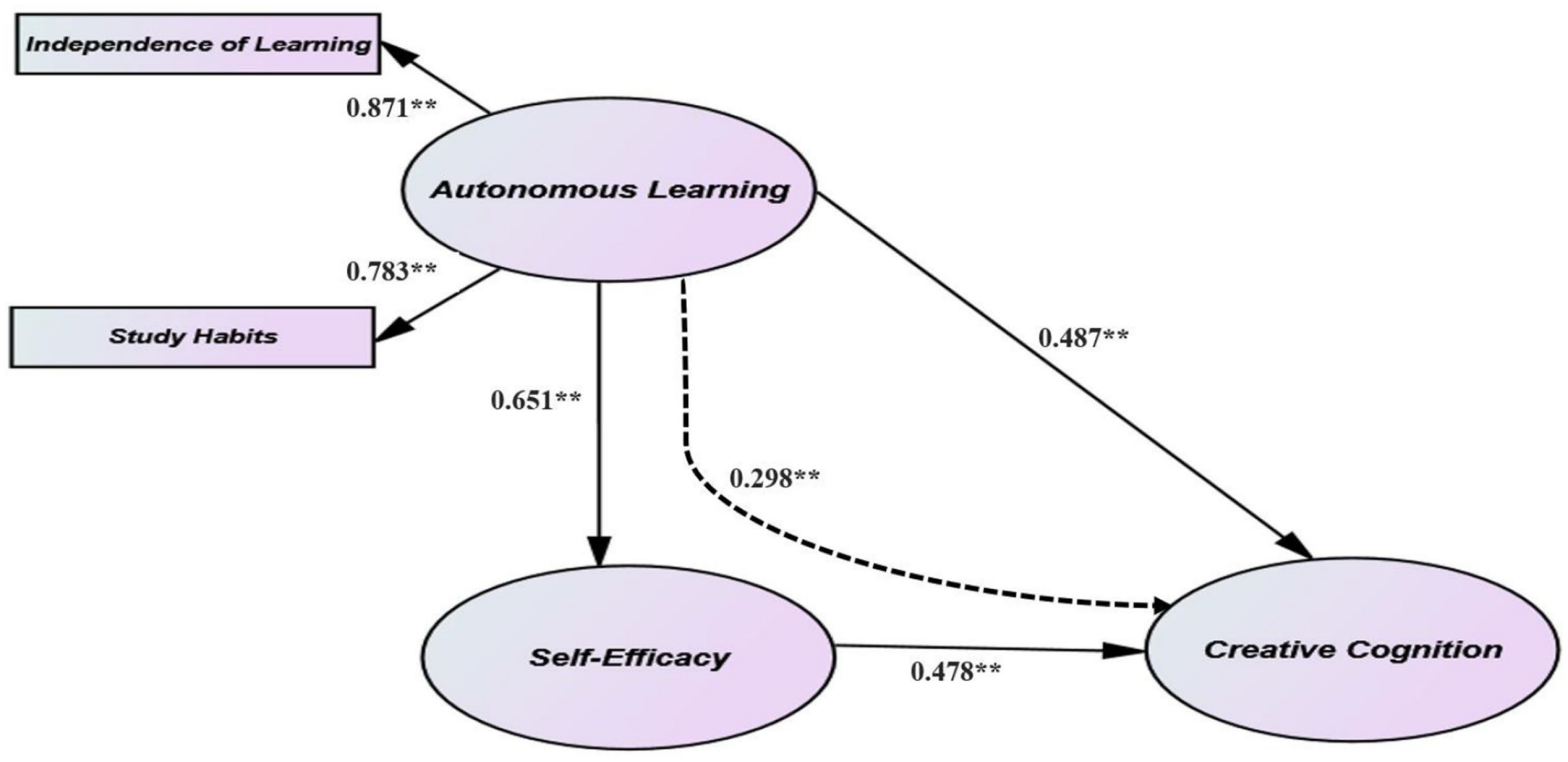
Results of the SEM analysis of the conceptual model of “autonomous learning” and “creative cognition,” *n* = 528. **p < 0.01.

The standardized parameter estimates illustrated in Figure 2 demonstrate that AL has a significant and direct impact on SE (*β* = 0.65, *p* < 0.01) and a moderate direct impact on CC (*β* = 0.49, *p* < 0.01). Additionally, SE has a moderately substantial direct impact on CC (*β* = 0.48, *p* < 0.01). Hence, the SEM outcomes confirm the first three hypotheses and offer preliminary support for the partial mediation model.

Subsequently, the Bootstrap analysis was conducted to investigate the significance and strength of meditation in the aforementioned relationships. Table 7 illustrates that the bootstrapping test outcomes supported SE’s significance as a mediator between AL and CC, confirming the fourth hypothesis. In addition, point estimates of impacts serve as an effect size indicator in the bootstrap analysis (Preacher and Hayes, 2008). Notably, as shown in Table 7, the total AL effect on CC amounted to 0.785. The direct effect of AL on CC was 0.435, with an indirect effect of 0.298 through SE, as shown in Table 7. It was revealed that about 38.87% of the total effect of AL on CC was indirect (i.e., mediated by SE), while about 61.13% was direct.

**Table 7:** Bootstrapping results for the effects of AL on CC through SE.

Variables	Point estimate	Product of coefficients		Bias corrected 95% CI		Percentile 95% CI		
		SE	Z	Lower	Upper	Lower	Upper	Two-tailed significance
Standardized total effect AL-CC	0.785	0.043	18.945	0.656	0.809	0.657	0.897	***
Standardized direct effect AL-CC	0.435	0.067	6.175	0.301	0.579	0.317	0.597	***
Standardized indirect effect AL-CC	0.298	0.041	8.923	0.259	0.309	0.210	0.367	***

*n* = 528, 2,000 bootstrap samples. ***p < 0.001.

In the following analyses, we investigated the interactions between the two dimensions of AL and other variables by scrutinizing the AL data. Henceforth, we utilized bootstrap analysis (Table 8) to scrutinize the association amid the two dimensions of AL, SE, and CC (Preacher and Hayes, 2008).

**Table 8:** Bootstrapping results for the standardized effects of the dimensions of AL on SE and CC.

		Product of coefficients		Bias corrected 95% CI		Percentile 95% CI		
Bootstrapping	Point estimate	SE	Z	Lower	Upper	Lower	Upper	Two-tailed Significance
IoL								
Total effect	0.345	0.088	4.276	0.181	0.463	0.189	0.482	***
Direct effect	0.205	0.075	1.345	0.159	0.276	0.124	0.267	***
Indirect effect	0.308	0.056	4.987	0.178	0.387	0.178	0.387	***
SH								
Total effect	0.329	0.098	4.316	0.189	0.461	0.183	0.484	***
Direct effect	0.209	0.081	1.381	0.154	0.289	0.144	0.287	***
Indirect effect	0.301	0.066	4.934	0.176	0.377	0.179	0.331	***

*n* = 528; 2,000 bootstrap samples. *p < 0.05; **p < 0.01; ***p < 0.001.

The results, as shown in Table 8, show that the first factor, IoL, had a moderate impact on CC, exhibiting a total effect size of 0.345. IoL significantly impacts CC indirectly (*β* = 0.308, *p* < 0.01) through SE, as verified by bootstrapping analysis, which entirely mediates the effects of IoL on CC. The second factor, SH, exhibited a moderate impact on CC, with a total effect size of 0.329. It has a noteworthy indirect effect on CC (*β* = 0.301, *p* < 0.01) via SE, which comprehensively mediates the impacts of SH on CC, confirmed by bootstrapping analysis.

In conclusion, the first and second dimension of AL, IoL, and SH, yielded substantial effects and act as an affective factor capable of significantly impacting CC. These findings provide evidence supporting Hypothesis five.

## 4 Findings and discussion

### 4.1 Learner autonomy, creative cognition, and self-efficacy

This study examined the correlations between students’ AL, SE and CC. Overall, the results indicate that AL and SE are important factors contributing to CC. In particular, the analysis process validates the applicability of the partial mediation model to the data provided. Thus, AL contributes to CC both directly and indirectly through activities associated with active AL and through shaping students’ SE. How AL affects CC is complicated, as seen by the interaction between direct and indirect pathways. The development of SE through AL activities not only fosters but also preserves creativity, even while the activities themselves immediately trigger it. As a result, the development of AL through active engagement and the enhancement of SE combine to provide a strong basis for innovative thinking. Based on an analysis of the research and discussions in the literature, it was hypothesised, and validated in the present study, that students’ AL characteristics increase their SE in relation to CC. The current study examined the effect of AL on students’ CC. The study found out that AL predicted CC positively and meaningfully. The significance and affirmative forecast of CC by AL highlights the need of promoting autonomy in learning environments. Teachers have the capacity to greatly increase students’ creative potential by assisting them in taking charge of their own education. This link emphasizes how important it is to incorporate AL activities into instructional tactics in order to foster both academic performance and the growth of the creative mind. Gaining knowledge of and using this predictive link can improve learning outcomes for students, improve instructional strategies, and eventually produce a more creative and adaptive student body. A large number of previous studies have shown a significant correlation between AL and CC (Çelik, 2017; Hashamdar and Rangriz, 2017; Nosratinia and Zaker, 2015; Shemirani et al., 2011). In this regard, Winch (2002) asserts that one aspect of psychological well-being that may influence student learning outcomes is autonomy. A link between learner autonomy and creative cognition is likely due to Ryff's (2013) emphasis on the fact that individuals with high levels of psychological well-being are satisfied, healthy, productive, and have rewarding interpersonal relationships.

Designing instructional interventions and support systems that optimize the direct and indirect advantages of autonomous learning requires an understanding of this dual effect. Teachers and policymakers must to take into account approaches that actively foster students’ self-efficacy growth in addition to autonomous learning activities. This all-encompassing method can better prepare students to use their creativity and realize their greatest potential.

In addition, the results of the current research showed that the participants with higher AL levels had higher mean CC scores. In light of this, it can be said that students with higher AL levels improved their creative ability. Therefore, this situation may favorably enhance their creative cognition.

The result that AL predicted SE was supported by similar studies (Cotterall and Crabbe, 1999; Ilgaz, 2011; Tılfarlıoğlu and Çiftçi, 2011). According to Bandura (1994), self-efficacy perceptions are fuelled by past experiences and observing the actions of successful people. In this respect, self-efficacy is also based on the past and positive situations in the past lead to an increase in self-efficacy. Individuals with high self-efficacy perceptions take more responsibility (Önen and Öztuna, 2005; Tatar et al., 2009). Learner autonomy is the learner’s sense of personal responsibility for learning (Holec, 1988). From this perspective, a student with a high belief in success and learning will also feel responsible for learning and have a high desire to learn which are conceptual components of learner autonomy.

The current study also discovered that SE was positively correlated with CC. The significance of SE as a driver of creative cognition is highlighted by the positive connection found between them. Individuals may improve their creative capacities and produce more original ideas and solutions by cultivating SE. This link emphasizes the necessity of organizational and educational strategies that foster and enhance SE in order to foster creativity and accelerate innovation. Comprehending this association offers significant perspectives on the ways in which confidence impacts creativity and shapes approaches to augment both personal and group creative potential. Most empirical research has also shown a strong correlation between self-efficacy beliefs and a variety of creative characteristics, such as being a creative person (Choi, 2004; Farmer, 2017; Gao, 2020; He, 2022; Laws, 2002; Seo et al., 2015). For instance, He (2022) found in her study that creative self-efficacy accounted for a significant amount of the variance (11%) in the likelihood of using creative thinking, and that the connection between self-efficacy and creative thinking tendency was of medium size. These findings provide evidence in favor of both the creative behavior and the social cognitive theory. In summary, a growing body of research has shown that self-efficacy has a positive influence on creativity (although conflicting results have been found, see Simmons et al., 2014). The relationship between self-efficacy and creative expression (the expression of ideas, solutions, processes and outcomes) has also been conceptualized and empirically demonstrated by research (e.g., Choi, 2004; Farmer, 2017). Based on these results, it can be concluded that SE and CC can promote each other. In other words, higher levels of self-efficacy among students can lead to greater improvements in their creative capacity.

The fourth hypothesis of this study investigates the effect of one intervening variable, SE, on the correlation between AL and CC. The mediator regulates the correlation between the dependent variable CC, and the independent variable AL. This study is the first to examine the correlation between the mediator, SE, and the domains of AL and CC. We reached this conclusion by scrutinizing a range of scholarly databases.

The study found that both dimensions of AL (IoL and SH) had a significant direct and indirect influence on CC through SE, validating the fifth hyphothesis that the dimensions of AL (IoL and SH) have distinct indirect impacts on CC through SE. The statement highlights the intricate relationships that exist between CC, SE, SH, and IoL. IoL and SH have different but complimentary functions in promoting SE, which in turn enhances CC constructively. Teachers and students can improve SE and creativity by implementing tailored methods to learning and study habits by having a clear understanding of these unique indirect consequences. Through identification and utilization of these distinct pathways, instructional strategies may be developed to facilitate independent learning as well as effective study habits, eventually enabling students to reach their full creative potential. A number of previous studies have also shown a significant association between AL and CC (Çelik, 2017; Hashamdar and Rangriz, 2017; Nosratinia and Zaker, 2015; Shemirani et al., 2011). In this regard, Winch (2002) asserts that one aspect of psychological well-being that may influence student learning outcomes is autonomy. A link between learner autonomy and creative cognition is likely due to Ryff's (2013) emphasis on the fact that individuals with high levels of psychological well-being are satisfied, healthy, productive, and have rewarding interpersonal relationships.

### 4.2 Implications (theory and practice)

The study theoretically advances our knowledge of self-efficacy and its function in the connection between autonomous learning and creative cognition, leading to more complex and complete models. From an educational, policy, and parental perspective, the results provide useful guidance on how to create conditions that facilitate autonomous learning, increase self-efficacy, and eventually improve creative cognition in gifted students. These implications emphasize how critical it is to incorporate theoretical understanding into real-world applications in order to promote the growth of creative and self-efficacious learners.

### 4.3 Limitations and suggestions for future research

The current study has also some limitations. One of the most important limitations of this study is that the study group was selected from only in Ankara, Türkiye. The study group also only consists of gifted secondary school students. In addition, the data was collected by means of three scales on which the students rated themselves. Therefore, it may contribute to the development of this study to collect the data with a study group that includes participants from a wider age range and different cultural backgrounds. The line of theoretical and empirical research that distinguishes autonomy from independence may be useful to consider in the present study. Little (1991) emphasizes that learner autonomy prioritizes “interdependence” above “independence” in the learning process, while Dickinson (1993) links autonomy to the concept of learning on its own and independence to taking active responsibility for one’s education. Lamb and Reinders (2006) propose that there exist “two strands of indepence / autonomy.” The first focuses on learning as a largely autonomous process, while the other one organizes learning to occur without the need for teacher supervision. Lamb and Reinders (2006) emphasize the “contextual nature of autonomy, and independence,” but they also contend that it is hard to come up with a single authoritative description that encompasses all the many perspectives on autonomy. Numerous studies, both theoretical and empirical, have looked closely at the differences between autonomy and independence, offering insightful information on their special traits and implications. Chirkov et al. (2003) have had a significant impact on the understanding of these ideas. Chirkov et al. (2003) defined autonomy as self-endorsed behaviors in which people believe that they are acting in a way that is consistent with who they really are. According to Chirkov et al. (2003), although “independence” refers to the capacity to function independently, it does not always include internalizing one’s own acts or coordinating them with personal ideals, which is essential to “autonomy.” In this study, including the present conceptual framework, methodology and the overall study, we have focused on autonomy.

The study also focused on the mediating role of SE on the relationship between AL and CC; the direct and indirect effects of different variables on the relationship between AL, CC, and SE can be analyzed in future studies. The present study found out that AL, SE and CC were found to be related to each other and to have a high and significant relationship among them. In this regard, these findings of the present study also suggest implications for raising qualified learners because the quality of a learner is positively expected to be influenced by the variables examined in the current study. The presence and quantity of qualified learners with high levels of creative cognition, self-efficacy and learner autonomy can also be expected to represent the qualified members of the society with higher-order thinking skills.

## 5 Conclusion

The present study demonstrated that AL positively and significantly predicted CC. SE was found to be positively correlated with CC. The study also found that both dimensions of AL (IoL and SH) had a significant direct and indirect influence on CC through SE, validating the hyphothesis that the dimensions of AL (IoL and SH) have distinct indirect impacts on CC through SE. These findings underscore the critical role of AL in enhancing CC, both directly and indirectly through SE. The study highlights the distinct pathways through which the dimensions of AL (IoL and SH) contribute to CC, thereby validating the hypothesis and emphasizing the importance of fostering AL to boost creative outcomes.

## Data Availability

The raw data supporting the conclusions of this article will be made available by the authors, without undue reservation.
