# Supplementary material for: Autonomous learning and creative cognition: the mediating effect of gifted students’ self-efficacy
Source: Front Psychol. 2025 Jan 3;15:1301528. doi: 10.3389/fpsyg.2024.1301528 (PMC11738625; doi:10.3389/fpsyg.2024.1301528)
Supplement: Supplementary file 1 [file Table_1.docx]

**Annex**

Scales used in this research:

**Autonomous Learning Scale**

| **Factor 1**  **Independence of Learning (n=528) (α=.81)** | **M** | **Sd** |
| --- | --- | --- |
| 1. I enjoy new learning experiences. | 3.19 | 1.52 |
| 1. I am open to new ways of doing familiar things. | 3.33 | 1.11 |
| 1. I enjoy being set a challenge. | 3.44 | 1.55 |
| 1. I enjoy finding information about new topics on my own. | 3.13 | 1.37 |
| 1. Even when tasks are difficult I try to stick with them. | 3.65 | 1.20 |
| 1. I tend to be motivated to work by assessment deadlines. | 3.59 | 1.03 |
| 1. I take responsibility for my learning experiences. | 3.09 | 1.41 |
| **Total** | 3.04 | 1.34 |
| **Factor 2**  **Study habits (n=528) (α=.80 )** |  |  |
| 1. My time management is good. | 3.06 | 1.49 |
| 1. I am good at meeting deadlines. | 3.87 | 1.20 |
| 1. I plan my time for study effectively. | 3.41 | 1.12 |
| 1. I frequently find excuses for not getting down to work. | 3.86 | 1.53 |
| 1. I am happy working on my own. | 3.67 | 1.07 |
| **Total** | 3.14 | 1.38 |

| **Creative Cognition Scale (n=528) (α=.75)** | **M** | **Sd** |
| --- | --- | --- |
| 1. I find effective solutions by combining multiple ideas. | 3.67 | 1.25 |
| 1. While working on something, I try to generate as many ideas as possible. | 3.93 | 1.51 |
| 1. I try to act out potential solutions to explore their effectiveness. | 3.26 |  |
| 1. If I get stuck on a problem, I try to take a different perspective of the situation. | 3.15 | 1.74 |
| 1. Incorporating previous solutions in new ways leads to good ideas. | 3.69 | 1.12 |
| **Total** | 3.24 | 1.54 |

| **Student Self-efficacy Scale (n=528) (α=.79)** | **M** | **Sd** |
| --- | --- | --- |
| 1. I am convinced that I am able to successfully learn all relevant subject content even if it is difficult. | 3.61 | 1.21 |
| 1. I know that I can maintain a positive attitude toward this course even when tensions arise. | 3.63 | 1.21 |
| 1. When I try really hard, I am able to learn even the most difficult content. | 3.16 |  |
| 1. I am convinced that, as time goes by, I will continue to become more and more capable of learning the content of this course. | 3.14 | 1.70 |
| 1. Even if I get distracted in class, I am confident that I can continue to learn well. | 3.19 | 1.22 |
| 1. I am confident in my ability to learn, even if I am having a bad day. | 3.21 | 1.10 |
| 1. If I try hard enough, I can obtain the academic goals I desire. 2. I am convinced that I can develop creative ways to cope with the stress that may occur while taking this course. 3. I know that I can stay motivated to participate in the course. 4. I know that I can finish the assigned projects and earn the grade I want, even when others think I can’t | 3.19  3.67  3.21  3.03 | 1.07  1.09  1.28  1.23 |
| **Total** | 3.24 | 1.54 |
